# High prevalence of sarcopenia among binge drinking elderly women: a nationwide population-based study

**DOI:** 10.1186/s12877-017-0507-3

**Published:** 2017-05-30

**Authors:** Jun-Il Yoo, Yong-Chan Ha, Young-Kyun Lee, Hana-Choi, Moon-Jib Yoo, Kyung-Hoi Koo

**Affiliations:** 1 Department of Orthopaedic Surgery, Gyeongsang National University Hospital, Jinju, Korea; 2 Department of Orthopaedic Surgery, Chung-Ang University College of Medicine, 102 Heukseok-ro, Dongjak-ku, Seoul, 156-755 South Korea; 3 Department of Orthopaedic Surgery and Rehabilitation, Dankook University College of Medicine, Cheonan, Korea

**Keywords:** Binge drinking, Alcohol consumption, Sarcopenia, Skeletal muscle

## Abstract

**Background:**

Alcohol consumption is considered a risk factor for sarcopenia, but the association between alcohol consumption and the prevalence of sarcopenia has not been evaluated in detail. This study was to identify the relationship between alcohol drinking patterns and the prevalence of sarcopenia in the elderly Korean population.

**Methods:**

The cross-sectional study was performed using data from the Korea National Health and Nutrition Examination Survey. Participants were excluded if they were under the age of 65, or if data was not available regarding skeletal muscle mass or dietary intake. After these exclusions, a total of 4020 participants (men: 1698; women: 2322) were analyzed in the present study. Sarcopenia is defined according to the criteria for the Asia Working Group for Sarcopenia (AWGS). Binge drinking was defined as consuming ≥5 standard alcoholic drinks (≥4 drinks for women) consecutively on one occasion. This data was subcategorized into two groups based on presence of binge drinking: Social drinking (≤1 time/month) and binge drinking (>1 time/month).

**Results:**

Women binge drinkers with weekly or daily consumption had 2.8 times higher prevalence of sarcopenia than social drinkers (Odds Ratio [OR] = 2.84; 95% Confidence Interval [CI] = 1.12–7.29). However, there were no associations between binge drinkers and sarcopenia in men. After adjusting for age, body mass index (BMI), energy intake, moderate physical activity, and energy intake, women binge drinkers with weekly or daily alcohol consumption had 3.9 times higher prevalence of sarcopenia than social drinkers (OR = 3.88; 95% CI = 1.33–11.36).

**Conclusions:**

The prevalence of sarcopenia in elderly women was related to binge drinking frequency and amounts of drinking after adjusting for covariates. Elderly Korean women who binge drink once or more per week may be associated with sarcopenia, as seen with the observed 3.9 times higher prevalence compared to social drinkers.

## Background

Habitual alcohol consumption is detrimental to health, although some beneficial effects have been documented [1, 2]. As an estimated 3.8% of all deaths and 4.6% of disability-adjusted life years lost are being attributed to alcohol consumption and alcoholism, excessive alcohol consumption is known to be a significant public health problem [3]. For Korean adults, alcohol drinking is a very common component of the social culture, and according to a nationwide database, 81.6% of adult men and 52.4% of women were reported to be alcohol drinkers [4]. The high prevalence of alcohol consumption is a current major health problem and socioeconomic burden in Korea [5].

Sarcopenia is defined as a loss of muscle strength and mass, and it generally results from a complex bone-muscle interaction in relation to chronic disease and aging [6]. In the elderly, sarcopenia is considering an independent risk factor for falls, disability, morbidity, and mortality [7–10]. Among several candidate risk factors for sarcopenia, alcohol consumption is known to be one of the risk factors due to the relationship of loss of skeletal muscle mass and alcohol consumption in animal studies [6, 11].

In human studies, although the relationship between alcohol consumption and sarcopenia has been reported in the general population, the number of studies are not large enough and their results are still controversial [12–14]. In addition, the results of a recently reported meta-analysis did not support alcohol consumption as a risk factor for sarcopenia [15]. However, studies included in that meta-analysis were not designed considering the relationship between alcohol consumption and sarcopenia as the primary end point.

Therefore, the purpose of this study was to identify the relationship between alcohol drinking patterns and the prevalence of sarcopenia in the elderly Korean population.

## Methods

### Participants

**Fig. 1:**
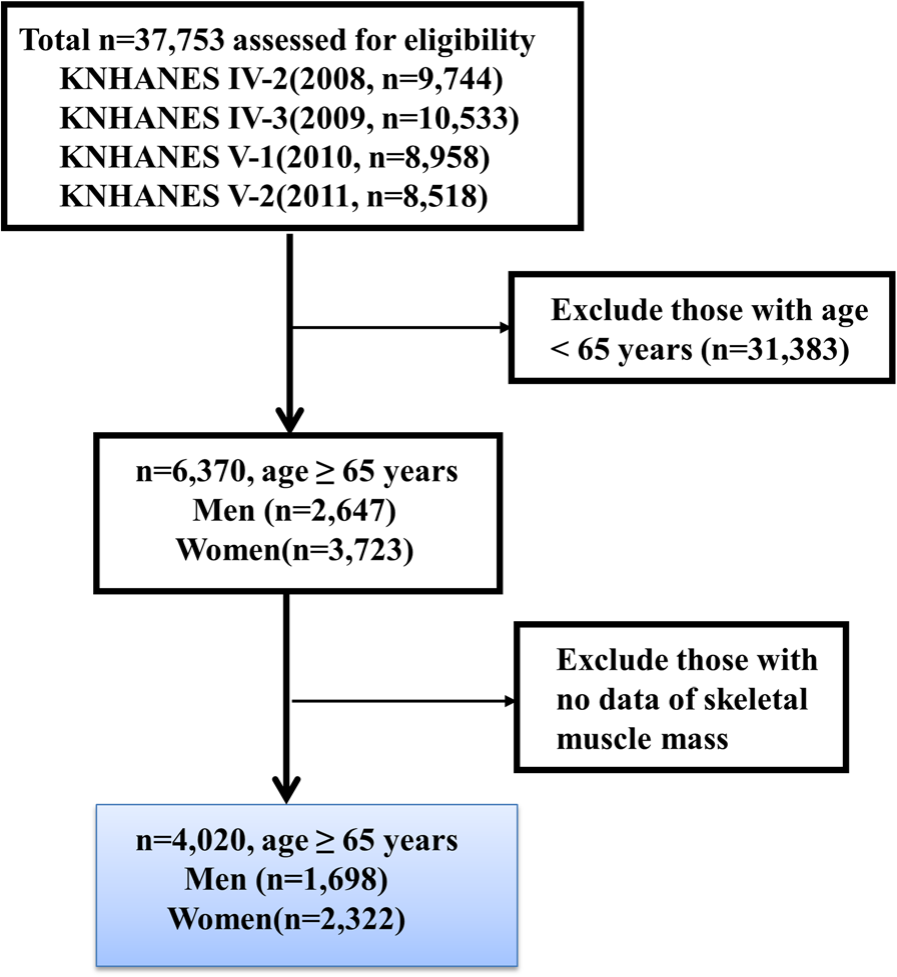
Selection process of study subjects, KNHANES IV, V (2008–2011)

This study was based on data from the 2008 to 2011 KNHANES, which was conducted by the Korea Ministry of Health and Welfare. KNHANES has been a nationwide representative cross-sectional survey for the Korean population with a clustered, multistage, stratified, and rolling sampling design. KNHANES consists of three sections: a health interview, a health examination, and a dietary survey. The survey data is collected via household interviews and by direct standardized physical examinations conducted in specially equipped mobile examination centers. The data was collected from 37,753 participants from survey years; 2008 (*n* = 9744), 2009 (*n* = 10,533), 2010 (*n* = 8958), and 2011 (*n* = 8518). Participants were excluded if they were under the age of 65, or if data was not available to evaluate skeletal muscle mass or dietary intake. After these exclusions, a total of 4020 participants (men: 1698; women: 2322) were analyzed for the present study (Fig. 1).

### Health examination survey

A health questionnaire was used to obtain information on age, gender, socioeconomic status, education status, smoking status (current, former, or never smoker), alcohol intake, moderate physical activity, and walking activity (yes or no). Moderate physical activity was defined as 5 or more days of moderately intense activity for at least 30 min per day. Walking physical activity was defined as 5 or more days of walking for at least 30 min per day. Body weight and height were measured in light clothing with no shoes, and body mass index (BMI) was calculated as weight (kg) divided by height squared (m^2^). Information regarding comorbidities, including: diabetes, chronic obstructive pulmonary disease (COPD), chronic renal failure (CRF), and malignancy as potential confounding factors were examined through the health interview survey.

### Drinking variables

The total amount of pure alcohol consumed (in grams per day) was calculated using the average number of alcoholic beverages consumed and the frequency of alcohol consumption. The participants were divided into three groups depending on the amount of alcohol consumed per day (non-drinker, light-to-moderate drinker [1–30 g/day], and heavy drinker [>30 g/day]). In this study, one glass was equivalent to roughly 8 g of pure alcohol, which can be found in 220 mL of regular beer of about 4.5% alcohol, and 50 ml of distilled spirits (e.g. Korean soju) of about 19% alcohol. The amount of alcohol was computed as: [amount of drink (mL) × volume of alcohol (%) × density of ethanol at room temperature (0.8)]/100. With 8 g of pure alcohol per glass, less than four glasses were considered equal to less than 32 g of pure alcohol. The frequency of drinking was divided into three groups: (< 1 time/month), (≥ 1 time/month and ≤2–4 times a month), (≥2–3 times a week).

Binge drinking was defined as consuming ≥5 standard drinks (≥4 drinks for women) consecutively in one occasion, and the data was subcategorized into two groups based on presence of binge drinking: Social drinking (≤1 time/month) and binge drinking (>1 time/month).

Alcohol Use Disorders Identification Test (AUDIT) scores were also considered. The participants were grouped according to their AUDIT scores: abstinence or low-risk drinking (0–7 points), more than low-risk drinking (8–14 points), harmful and hazardous drinking (15–19 points), and alcohol dependence (>20 points) [16].

### Measurements of appendicular skeletal muscle mass and definition of sarcopenia

Body composition in the KNHANES was measured by whole-body dual x-ray absorptiometry (DXA) (QDR 4500A, Hologic, Inc., Waltham, MA, USA). All subjects changed into paper gowns and were asked to remove all jewelry and other personal effects that could interfere with the DXA examination. To obtain accurate and reliable results, all data regarding body composition were gathered by educated and quality controlled sarcopenia examination surveyors [17]. Bone mineral content, fat mass, and lean soft-tissue mass were measured separately for each part of the body, including the arms and legs. The lean soft-tissue masses of the arms and legs were nearly equal to the skeletal muscle mass. As absolute muscle mass correlates with height, the skeletal muscle mass index was calculated by the following formula: (lean mass [kg]/height [m]^2^), which is directly analogous to body mass index (BMI = weight [kg]/height [m]^2^). Arm skeletal muscle mass index was defined as (arm lean mass [kg]/height [m]^2^). Leg skeletal muscle mass index was defined as (leg lean mass [kg] /height [m]^2^). Appendicular skeletal muscle mass index (SMI) was defined as the sum of the arm and leg SMIs. Sarcopenia was defined according to the criteria for the Asia Working Group for Sarcopenia (AWGS) (SMI of below 5.4 kg/m^2^ in women and below 7.0 kg/m^2^ in men) [18].

### Dietary intake measurement

Dietary intake was assessed by trained staff using a complete 24-h recall method. Daily intake of energy and protein was calculated by referencing nutrient concentrations in foods according to the Korean Food Composition Table.

## Statistical analysis

Complex sample analyses were used in this study to correct for the distributions of the cluster samples regarding the primary sampling unit, covariance and significance to correspond with those of the general Korean population. The target population for the sampling procedure in the Korean National Health and Nutrition Examination Survey was residents of Korea. However, residents of nursing homes, military facilities, and prisons were excluded. In order to improve the accuracy of the nationwide representative data, the sample design was carried out in a three-year cycle (according to the time of the year). Also, a survey of household members was conducted for the sample area and the number of residential households extracted through the sample design. We selected 20 households as the sample size. All analyses were carried out with the sample weights of KNHANES.

A rolling sampling survey refers to a survey in which independent rolling samples (n = F), not overlapping with the entire sample, are established and compared. In this case, each rolling sample should be extracted in the way that the probability distribution should be the sampling ratio of f = 1/F for the entire sample. Therefore, at the time of passing the F ^th^ cycle, the cumulative of the samples surveyed for all the time will organize a sample survey for the entire population.

In order to compare means between the non-sarcopenia group and the sarcopenia group, the Student’s t-test was used. To compare proportions, the chi-squared (χ^2^) test was used, and univariate analysis was performed. To differentiate interaction between chronic disease and binge drinking for sarcopenia, multivariable analysis including interaction term was conducted.

Variables that had a *p* value of <0.10 were included in the multivariate model. Multiple logistic regression analysis was conducted to calculate ORs and 95% CIs for the association between the frequency of binge drinking and the presence of sarcopenia after the adjustment of demographic variables (age, BMI, smoking status, moderate PA, and energy intake) served as covariates. All statistical tests were two-tailed, and statistical significance was defined as *p* < 0.05. The statistical calculation was performed using SPSS Statistics V.22 (SPSS, Chicago, IL, USA).

## Results

### Characteristics of elderly Korean population older than 65 years by presence of sarcopenia

In the elderly men, age (*p* = 0.006), chronic renal failure (*p* = 0.01), and malignancy (*p* < 0.001) were significantly higher in the sarcopenia group than the non-sarcopenia group. Although former cigarette smoking status was significantly higher in the sarcopenia group, current smoking status was significantly lower (*p* = 0.012). Waist circumference (*p* < 0.001) and moderate physical activity (*p* = 0.043) were significantly lower in the sarcopenia group than the non-sarcopenia group (Table 1).

**Table 1:** Characteristics of elderly Korean men older than 65 years by presence of sarcopenia

Variables	All	Non-sarcopenia	Sarcopenia	*P*-value
	(*n* = 1698)	(*n* = 1190)	(*n* = 508)	
Age (years)*	72 ± 5.2	71.3 ± 4.9	73.8 ± 5.4	0.006
BMI (kg/m^2^)*	23.1 ± 2.9	23.9 ± 2.6	20.9 ± 2.7	0.232
Waist circumference (cm)*	84.5 ± 9.1	86.6 ± 8.2	79.4 ± 9.2	<0.001
Appendicular SMI (kg/m^2^)*	7.4 ± 1.0	7.9 ± 0.6	6.3 ± 0.6	0.092
Education (%)				0.368
< Elementary school	49.7	48.8	51.8	
Elementary school	17.2	18.2	15.0	
Middle school	18.8	19.2	17.7	
> Middle school	12.5	12.4	12.8	
Cigarette smoking status (%)				0.012
Never	16.7	17.7	14.4	
Former	25.0	23.2	29.3	
Current	56.9	58.2	54.1	
Moderate physical activity (%)^a^	12.2	13.4	9.6	0.043
Walking physical activity (%)^b^	53.2	54.6	50.0	0.161
Income (%)				
Quartile 1 (lowest)	24.5	23.4	27.2	0.302
Quartile 2	23.9	24.3	23.0	
Quartile 3	24.7	24.6	25.0	
Quartile 4 (highest)	25.1	26.1	23.0	
Energy intake (kcal/day)*	1890.9 ± 699.3	1956.7 ± 695.6	1735.4 ± 684.0	<0.001
Protein intake (g/day)*	63.1 ± 31.2	65.7 ± 31.8	56.7 ± 28.6	<0.001
Comorbidity (%)				
Diabetes	17.2	16.7	19.5	0.118
Chronic obstructive pulmonary disease	0.4	0	1.2	0.349
Chronic renal failure	0.6	0.5	0.8	0.01
Malignancy	6.5	5.2	9.6	<0.001

^*^All values are presented as the mean ± SD and percentage distribution of participants as appropriate: *BMI* body mass index, *SMI* skeletal muscle index, *BMD* bone mass density. Significance was compared between non-sarcopenia and sarcopenia groups using Student’s t-test or Pearson chi-square test

^a^Moderate physical activity was 5 or more days of moderate-intensity activity of at least 30 min per day

^b^Walking physical activity was 5 or more days of walking of at least 30 min per day

In the elderly women, age was significantly higher in the sarcopenia group than the non-sarcopenia group (*p* < 0.001). However, BMI (*p* < 0.001), waist circumference (*p* < 0.001), appendicular SMI (*p* < 0.001), energy intake (*p* < 0.001), and protein intake (*p* = 0.001) were significantly lower in the sarcopenia group than the non-sarcopenia group (Table 2).

**Table 2:** Characteristics of elderly Korean women older than 65 years by presence of sarcopenia

Variables	All	Non-sarcopenia	Sarcopenia	*P*-value
	(*n* = 2322)	(*n* = 2071)	(*n* = 251)	
Age (years)*	72 .5 ± 5.5	72.3 ± 5.4	74.2 ± 6.3	<0.001
BMI (kg/m^2^)*	24.1 ± 3.4	24.5 ± 3.3	20.9 ± 2.5	<0.001
Waist circumference (cm)*	83.1 ± 9.7	84.0 ± 9.5	76.1 ± 8.8	<0.001
Appendicular SMI (kg/m^2^)*	6.4 ± 1.1	6.5 ± 1.0	5.0 ± 0.3	<0.001
Education (%)				0.499
< Elementary school	86.7	87.0	84.2	
Elementary school	6.3	6.2	7.5	
Middle school	5.4	5.2	7.1	
> Middle school	1.6	1.6	1.2	
Cigarette smoking status (%)				0.245
Never	89.6	89.9	86.6	
Former	1.6	1.5	2.4	
Current	8.8	8.6	10.9	
Moderate physical activity (%)^a^	12.3	12.8	8.6	0.064
Walking physical activity (%)^b^	39.7	40.0	38.0	0.561
Income (%)				0.711
Quartile 1 (lowest)	24.4	24.5	23.8	
Quartile 2	25.5	25.9	22.9	
Quartile 3	24.4	24.3	25.8	
Quartile 4 (highest)	25.6	25.4	27.5	
Energy intake (kcal/day)*	1420.6 ± 534.5	1437.6 ± 540.7	1276.1 ± 454.0	<0.001
Protein intake (g/day)*	44.7 ± 22.9	45.3 ± 23.2	40.0 ± 19.5	0.001
Comorbidity (%)				
Diabetes	17.7	17.8	17.0	0.352
Chronic obstructive pulmonary disease	0.5	0.5	0	0.892
Chronic renal failure	0.7	0.7	0.8	0.053
Malignancy	5.7	5.6	6.4	0.641

^*^All values are presented as the mean ± SD and percentage distribution of participants, as appropriate: *BMI* body mass index, *SMI* skeletal muscle index, *BMD* bone mass density. Significance was compared between non-sarcopenia and sarcopenia groups using Student’s t-test or Pearson chi-square test

^a^Moderate physical activity was 5 or more days of moderate-intensity activity for at least 30 min per day

^b^Walking physical activity was 5 or more days of walking of at least 30 min per day

### Pattern of alcohol consumption according to the prevalence of sarcopenia

Table 3 shows subject characteristics of alcohol consumption according to the prevalence of sarcopenia. For the elderly men, drinking quantity, binge drinking, and AUDIT scores, there were no significant differences between the sarcopenia and non-sarcopenia groups. However, drinking frequency was more frequent in the non-sarcopenia group than the sarcopenia group (*p* = 0.011).

**Table 3:** Patterns of alcohol consumption according to the prevalence of sarcopenia

Variables	Men (*n* = 1698)	Non-sarcopenia (*n* = 1190)	Sarcopenia (*n* = 508)	*P*-value	Women	Non-sarcopenia	Sarcopenia	*P*-value
Quantity of Drinking (%)				0.906				0.022
Non-drinker	266 (15.7)	184 (15.5)	82 (16.1)		74.4	74.0	77.5	
Light-to-moderate drinker	1168(68.8)	82 (68.7)	351 (69.1)		25.2	25.8	20.9	
Heavy drinker	264 (15.5)	188 (15.8)	75 (14.8)		0.3	0.2	1.6	
Frequency of drinking (%)				0.017				0.366
< 1 time/month	773 (45.5)	515 (43.3)	258 (50.8)		68.9	68.3	72.9	
≥ 1 time/month and ≤2–4 times a month	522 (30.7)	382 (32.1)	140 (27.6)		21.9	22.5	17.1	
≥ 2–3 times a week	403 (23.7)	293 (24.6)	110 (21.7)		9.3	9.2	10.1	
Precense of binge drinking (%)				0.233				0.024
Social drinker	1224 (72.1)	841 (70.7)	385 (75.8)		96.3	96.9	91.5	
Binge drinker	474 (27.9)	349 (29.3)	123 (24.2)		3.7	3.1	8.5	
AUDIT score (%)				0.134				0.311
0 to 7	883 (52.0)	594 (49.9)	293 (57.7)		89.6	96.3	93.0	
8 to 14	465 (27.4)	341 (28.7)	123 (24.2)		1.6	3.1	6.2	
15 to 19	199 (11.7)	147 (12.4)	49 (9.7)		8.8	0.1	0.0	
> 20	151 (8.9)	108 (9.1)	43 (8.4)		12.3	0.6	0.8	

In elderly women, the frequency of drinking and AUDIT scores revealed no significant differences between the sarcopenia and non-sarcopenia groups. Although the quantity of alcohol consumption in heavy drinkers was larger in the sarcopenia group than the non-sarcopenia group, the quantity of alcohol consumption in light-to-moderate drinkers was larger in the non-sarcopenia group than the sarcopenia group (*p* = 0.022). Also, binge drinking was more frequent in the sarcopenia group than the non-sarcopenia group (*p* = 0.024).

### Univariate analysis of the variables for sarcopenia

In elderly men, age (*p* < 0.001), body mass index (*p* < 0.001), waist circumference (*p* < 0.001), former cigarette smoker (*p* = 0.011), moderate physical activity (*p* = 0.04), energy intake (*p* < 0.001) and protein intake (*p* < 0.001) were significantly affected by sarcopenia. For elderly women, age (*p* < 0.001), body mass index (*p* < 0.001), waist circumference (*p* < 0.001), moderate physical activity (*p* = 0.066), energy intake (*p* < 0.001) and protein intake (*p* = 0.001) were significantly affected by sarcopenia. Chronic renal failure, diabetes and smoking status with binge drinking in men and women were not significantly affected by sarcopenia as interaction term.

### Odds ratios for sarcopenia according to alcohol drinking patterns in the elderly population

The odds ratios for the prevalence of sarcopenia according to alcohol drinking patterns are shown in Table 4. In the elderly men, after adjusting for age, body mass index, smoking status, moderate physical activity, and energy intake, odds ratios of sarcopenia according to alcohol drinking patterns displayed no statistically significant differences.

**Table 4:** Odds ratios (ORs) and 95% confidence intervals (CIs) for sarcopenia according to alcohol-drinking patterns in elderly population

	Men				Women			
Variables	Unadjusted	*P*-value	Adjusted^a^	*P*-value	Unadjusted	*P*-value	Adjusted^a^	*P*-value
	OR (95% CI)		OR (95% CI)		OR (95% CI)		OR (95% CI)	
Quantity of Drinking (%)								
Non-drinker	1 (reference)		1 (reference)		1 (reference)		1 (reference)	
Light-to-moderate drinker	0.93 (0.64–1.36)	0.71	0.93 (0.59–1.47)	0.743	10.22 (1.38–75.49)	0.023	5.45 (0.50–59.36)	0.16
Heavy drinker	0.73 (0.50–1.08)	0.12	0.87 (0.54–1.39)	0.548	7.93 (1.11–56.92)	0.04	4.42 (0.42–46.75)	0.22
Frequency of drinking (%)								
< 1 time/month	1 (reference)		1 (reference)		1 (reference)		1 (reference)	
≥ 1 time/month and ≤2–4 times a month	0.75 (0.58–0.97)	0.03	0.81 (0.59–1.11)	0.194	1.03 (0.56–1.92)	0.92	0.74 (0.37–1.49)	0.39
≥ 2–3 times a week	0.65 (0.48–0.88)	0.001	1.03 (0.71–1.48)	0.881	0.71 (0.44–1.16)	0.17	0.90 (0.52–1.54)	0.69
Presence of Binge drinking (%)								
Social drinker	1 (reference)		1 (reference)		1 (reference)		1 (reference)	
Binge drinker	0.77 (0.76–1.05)	0.09	0.95 (0.66–1.38)	0.791	2.84 (1.12–7.29)	0.03	3.88 (1.33–11.36)	0.01

^a^Adjusted for age, body mass index, smoking status, moderate physical activity, and energy intake in men and women

For elderly women, for quantity of drinking, after adjusting for age, body mass index, moderate physical activity, and energy intake, odds ratios for the sarcopenia versus the non-sarcopenia were no statistically different. However, elderly women binge drinkers with a weekly or daily consumption had 2.8 times higher prevalence of sarcopenia than social drinkers (OR = 2.84; 95% CI = 1.12–7.29). After adjusting for age, body mass index, moderate physical activity, and energy intake, binge drinkers with weekly or daily consumption had 3.9 times higher prevalence of sarcopenia than social drinkers (OR = 3.88; 95% CI = 1.33–11.36).

## Discussion

Although alcohol consumption has been proven to cause muscle atrophy in animal studies, the relationship between alcohol consumption and sarcopenia in humans is still controversial [19–22]. This nationwide cross-sectional study identified the relationship between sarcopenia and alcohol drinking pattern in elderly women. After adjusting for variables, it was found that among elderly women, binge drinkers with weekly or daily consumption had a 3.9 times higher prevalence of sarcopenia than social drinkers.

So far, several basic experimental animal studies have proven the relationship between alcohol consumption and sarcopenia. A few studies were performed to assess the net effect of alcohol consumption on the prevalence of sarcopenia. These studies reported that acute alcohol consumption decreased muscle protein synthesis and caused changes in skeletal muscles in female mice and rats [19, 20]. Human studies reported that heavy alcohol consumption in patients with alcoholic cirrhosis was related to acceleration of sarcopenia [23, 24]. However, these findings are not consistent with those of other human studies. The results of a meta-analysis to explore the relationship between sarcopenia and alcohol consumption did not support alcohol consumption as a risk factor for sarcopenia [15]. However, the recent meta-analysis study has several important limitations. Among the references of the study, there was no study that considered the relationship between sarcopenia and alcohol consumption as the primary end point. In addition, the study did not confirm the relationship between alcohol drinking pattern and sarcopenia [15]. This study was primarily designed to assess the relationship between sarcopenia and alcohol drinking pattern. In the current study, among elderly women, binge drinkers with weekly or daily consumption had a 3.9 times higher prevalence of sarcopenia than social drinkers (OR = 3.88; 95% CI = 1.33–11.36) after adjusting for age, body mass index, moderate physical activity, and energy intake and after considering interaction terms such as chronic renal failure, diabetes and smoking status.

Although we could not identify the relationship between alcohol consumption and sarcopenia among men, among elderly women, binge drinking was related to sarcopenia. Elderly women who binge drink are more vulnerable to the consequences of alcohol consumption than men, as proven in other studies [25–28]. The possible related mechanisms that might be involved are as follows: women have proportionately more body fat and a lower volume of body water compared with men of similar weight [29]. As a result, women have a higher concentration of alcohol because there is less volume of water to dilute the alcohol. In addition, elderly women have even less body water, a decreased tolerance for alcohol, and an even slower metabolism rate for alcohol [30]. Therefore, sarcopenia associated with the metabolic disorder may also be affected by gender differences in alcohol’s effects.

This study has several limitations. First, it could not evaluate the causality between alcohol consumption and low skeletal muscle mass. Prospectively designed studies are necessary to clarify this relationship. Second, we classified the subjects into three alcohol-drinking groups based on a self-reported questionnaire. The use of self-reported information on alcohol-drinking patterns could lead to misrepresentation of actual drinking styles. Third, self-reported alcohol intake, AUDIT scores, and smoking status may be underreported due to recall and social desirability biases. Finally, we adopted different cut-off levels of binge drinking in men and women. We that said, we could not adopt different cut-off levels of quantity of drinking in men and women. The reason of this limitation was that in the survey the quantity of drinking was not separately analyzed for men and women. In addition, the definitions of alcoholism according to DSM-V and WHO guidelines were not used to quantify the extent of drinking in men and women.

## Conclusions

In conclusion, the prevalence of sarcopenia in elderly Korean women was related to binge drinking frequency and amount of drinking after adjusting for covariates. Korean women who binge drink once per week or more than one time per week have almost 4 times higher risk of sarcopenia than female social drinkers.

## Abbreviations

ALP: Alkaline phosphatase
AUDIT: Alcohol Use Disorders Identification Test
AWGS: Asia Working Group for Sarcopenia
BMI: Body mass index
CI: Confidence interval
COPD: Chronic obstructive pulmonary disease
CRF: Chronic renal failure
DXA: Dual x-ray absorptiometry
KCDC: Korea Centers for Disease Control and Prevention
KNHANES: Korean National Health and Nutrition Examination Survey
ORs: Odds ratios
PTH: Parathyroid hormone
SMI: Skeletal muscle mass index
